# Variation of bending rigidity with material density: bilayer silica with nanoscale holes

**DOI:** 10.1039/d2cp01960d

**Published:** 2022-06-06

**Authors:** Martin Tømterud, Sabrina D. Eder, Christin Büchner, Markus Heyde, Hans-Joachim Freund, Joseph R. Manson, Bodil Holst

**Affiliations:** Department of Physics and Technology, University of Bergen Allégaten 55 5007 Bergen Norway martin.tomterud@uib.no; Fritz-Haber-Institut der Max-Planck-Gesellschaft Faradayweg 4-6 14195 Berlin Germany; Department of Physics and Astronomy, Clemson University Clemson South Carolina 29634 USA; Donostia International Physics Center (DIPC) Paseo Manual de Lardizabal, 4 20018 Donostia-San Sebastián Spain

## Abstract

Two dimensional (2D) materials are a young class of materials that is foreseen to play an important role as building blocks in a range of applications, *e.g.* flexible electronics. For such applications, mechanical properties such as the bending rigidity *κ* are important. Only a few published measurements of the bending rigidity are available for 2D materials. Nearly unexplored is the question of how the 2D material density influences the bending rigidity. Here, we present helium atom scattering measurements on a “holey” bilayer silica with a density of 1.4 mg m^−2^, corresponding to 1.7 monolayers coverage. We find a bending rigidity of 6.6 ± 0.3 meV, which is lower than previously published measurements for a complete 2D film, where a value of 8.8 ± 0.5 meV was obtained. The decrease of bending rigidity with lower density is in agreement with theoretical predictions.

## Abstract 2

Helium atom scattering experiments on a “holey” silica bilayer film reveal that the bending rigidity of the material, extracted from phonon dispersion curves, decreases with decreasing material density.

## Graphical abstract

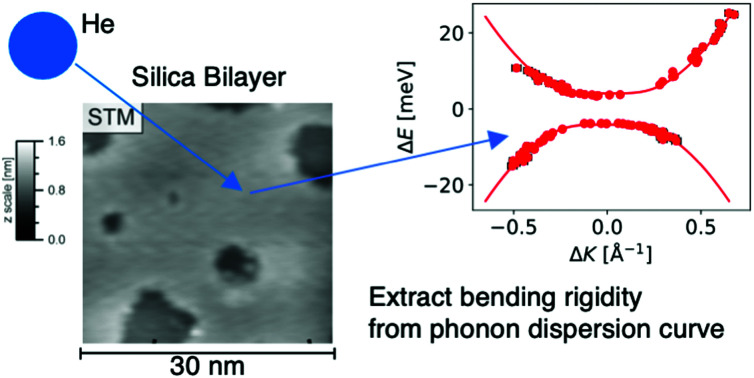

The bending rigidity of a material, sometimes also referred to as the bending modulus, expresses the flexibility of the material and is an essential metric for technological applications, such as flexible electronics^1^ and van der Waals materials. For 2D materials, the bending rigidity has proven challenging to measure.^2^

Helium atom scattering (HAS) is a uniquely well-equipped technique for characterising surfaces and 2D materials. This suitability is due to the combination of its neutral, non-penetrating nature and low incident energies, in addition to wavelengths comparable to interparticle separation.^3–5^ Al Taleb *et al.* showed that inelastic HAS can be applied to measure the bending rigidity of 2D materials.^6^ By measuring the dispersion of the out-of-plane flexural mode, also referred to as the bending mode, or ZA-mode, they extracted the bending rigidity of monolayer graphene grown on copper foil. Applying HAS in this manner has the advantage that the 2D material does not have to be free-standing; it can be weakly bound to a substrate. The substrate influences the lowest-energy phonon modes if the material is too tightly bound. In particular, one expects to find evidence of, and possible interference with, the Rayleigh mode of the substrate.^7^

In 2018, HAS was applied to measure the bending rigidity of 2D silica.^8,9^ The results were found to be in reasonable agreement with computations of crystalline 2D silica.^10^ Bilayer silica is a particularly interesting 2D material because it is a wide bandgap material,^11^ it can be used as an insulating material in material stacks,^12^ and experiments have demonstrated the potential of this 2D material to act as both a defined atomic sieve layer^13^ and a capturing device for noble gas atoms.^14^ Furthermore, bilayer silica allows for large scale transfer from its original growth substrate to another single crystal surface.^15^ This, together with recent advances demonstrating that bilayer silica can be grown fast and on large scale using atomic layer deposition (ALD) methods,^16,17^ makes bilayer silica an application-ready material. It is therefore of particular interest to document its mechanical properties.

Very recently, some of the present co-authors applied HAS to measure the temperature dependence of the bending rigidity of AB-stacked bilayer graphene,^18^ explicitly showing that bending rigidity increases with temperature. In the present work, we pose the question of how decreasing the mass density of the sample affects the bending rigidity, and we apply inelastic HAS to measure the bending rigidity of a silica bilayer film with nanoscale holes.

It was shown many years ago that the long wavelength (*i.e.* short momentum transfer) dispersion relation of the ZA-mode depends on the bending rigidity, *κ*.^19^ For a large, thin, freestanding membrane with fixed edge (d’Alembertian) boundary conditions, one obtains a dispersion relation as a function of parallel wave vector transfer Δ**K** given by^20,21^1
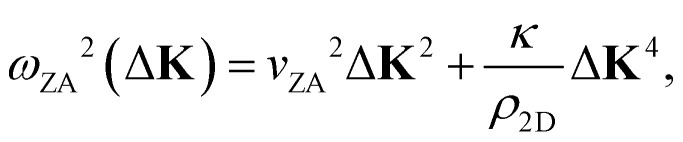
where *ρ*_2D_ is the 2D mass density and 
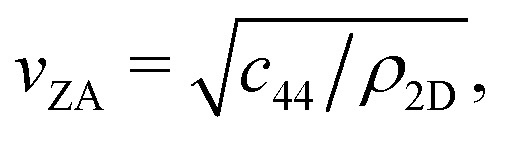
 where *c*_44_ is the shear force constant of the material. A few years ago Amorim and Guinea showed that when a thin membrane is weakly coupled to a substrate, a gap *ω*_0_, is introduced, where *ω*_0_ is the binding energy between the substrate and the membrane.^22^ The dispersion relation for the ZA-mode after this inclusion reads as2
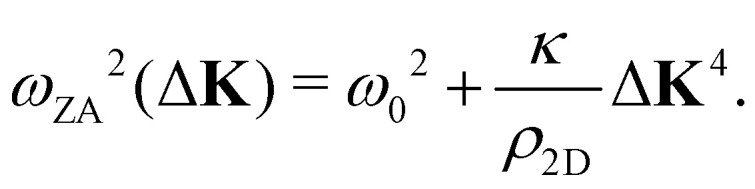
In principle, a quadratic acoustic term is also present in eqn (2); however, the constant term dominates it at small Δ**K**, and it is, therefore, negligible.^9^

The 2D silica sample used for the experiments presented in this paper was prepared at the Fritz-Haber Institut in Berlin in ultra-high vacuum (UHV) on a substrate of Ru(0001). The sample was investigated using scanning tunnelling microscopy (STM) and low-energy electron diffraction (LEED). The film exhibited crystalline and vitreous silica film areas, with both diffraction peaks and a ring visible in LEED. After preparation in Berlin, the sample was transported to Bergen, during which time it was exposed to ambient conditions for more than 20 hours. The sample was installed in an argon vented sample chamber upon arriving in Bergen and pumped down. The background pressure of the chamber was approximately 1 × 10^−9^ mbar. Before measurements, the sample was cleaned by heating to *T*_S_ = 675 K for 1 h under a partial O_2_ oxygen pressure of *p*_O_2__ = 2.2 × 10^−6^ mbar. The partial O_2_ pressure was turned off at *T*_S_ < 400 K during the cooling process of the sample. HAS experiments were carried out in the molecular beam apparatus, named MAGIE, at the University of Bergen.^23,24^ The neutral helium beam was created by a free-jet expansion from a source reservoir through a diameter 10 ± 1 μm nozzle. A skimmer, 410 ± 2 μm in diameter, placed 17.5 ± 0.5 mm in front of the nozzle, selected the central part of the beam. The distance between the chopper and the sample was 1.003 m, with a total nozzle-to-sample distance of 1.546 m, and sample-to-detector distance 0.905 m. The stagnation pressure in the source, *p*_0_, was between 70–80 bar. The measurements were conducted with the limiting aperture placed between the sample and the chopper chambers, 1.325 m from the nozzle giving a beamwidth of 7 mm on the sample. The detector opening was 4.6 mm wide. Over several days, a slight decrease in the reflected signal intensity could be observed. The original intensity was restored by repeating the cleaning process described above. The source-detector angle was kept fixed at 90°. All experiments were carried out with a room temperature sample *T*_S_ = 296 ± 2 K. Diffraction measurements were taken with a room temperature beam, which corresponds to a beam energy of approximately *E*_0_ = 64 meV. Time-of-flight (TOF) measurements were performed with a cooled beam of about *E*_0_ = 29 meV. With the given geometry of the experimental apparatus, this gives a coherence length of about 100 Å.^25^ The measurements presented were conducted in three independent series at three different azimuthal angles *ϕ* in order to check that the results were isotropic. The incident beam angle *θ*_i_ was varied during the series. Before the measurements started, the sample structure was characterised by in-plane diffraction curves, shown in Fig. 1. Two different azimuthal angles are investigated, with *ϕ* = 0.0° corresponding to the *ΓM* direction of the Ru(0001) substrate. The signs of a vitreous ring structure are visible with peaks at about 36° and 54°.

**Fig. 1:**
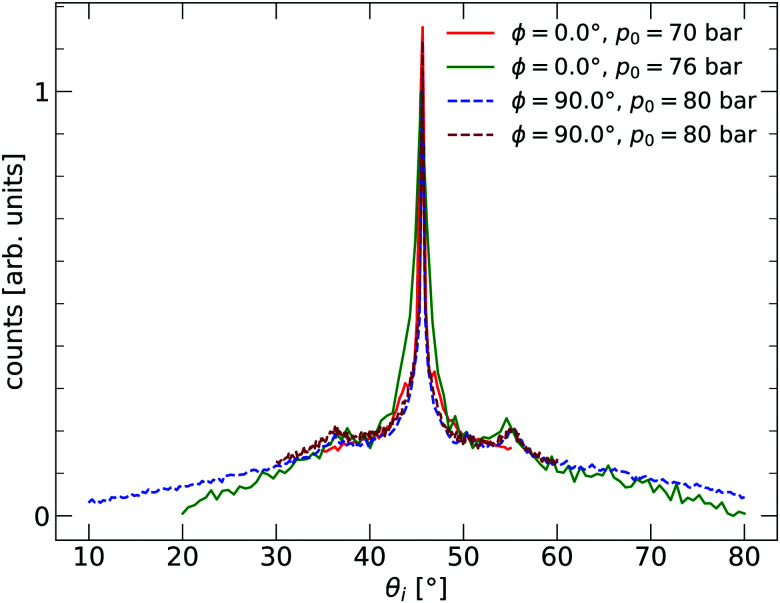
Angular distribution curves of the bilayer silica film with nanoscale holes supported on Ru(0001). Taken with a beam energy of *E*_i_ = 28.8 meV. *ϕ* = 0.0° corresponds to the *ΓM* direction of the substrate. The two curves labelled with *ϕ* = 90.0° are independent runs.

The local area density for the silica bilayer can vary between 1.4 mg m^−2^ and 1.8 mg m^−2^ depending on the ring size distribution and local arrangement in crystalline and amorphous film areas.^8,26^ The average area density for the silica bilayer film system can be estimated to be 1.6 mg m^−2^. An elegant way to tune the silica bilayer film properties is to change the overall monolayer (ML) coverage of the film system across the sample area. The silica film can be grown in a closed bilayer (2 ML) or a “holey” film structure (<2 ML). For samples with initial coverage between 1.6–1.8 ML, most of the holes have been reported to have an area of up to 10–40 nm^2^,^27^ corresponding to a hole diameter less than 3.5 nm. The change in the coverage allows for modifying the overall average area mass density. The open holes in the silica film have been characterised by STM images, an example of which can be seen in Fig. 2, together with a model of the bilayer. Altogether 8 STM images of various sizes (ranging from 30 nm × 30 nm to 50 nm × 50 nm), were examined. After determining the coverage of each one and taking a weighted average, we obtain an average coverage of 1.7 ± 0.1 ML; *i.e.* about 15% holes. The area mass density of this sample is therefore determined to be *ρ*_2D_ = 1.4 ± 0.1 mg m^−2^. We want to make the comments that some holes may, in fact, be filled by a single ML silica film^27^ and not go all the way through as indicated in Fig. 2. If a hole is filled by a silica ML, there will be pinning to the substrate in that area. However, the pinning implies that the density of the free-swinging part of the bilayer will be the same regardless of whether the holes are empty or covered by a ML. Furthermore, if the silica ML inside the holes played a dominant role compared to the open holes, this should be visible as a Rayleigh mode in the phonon dispersion curve. See discussion further on.

**Fig. 2:**
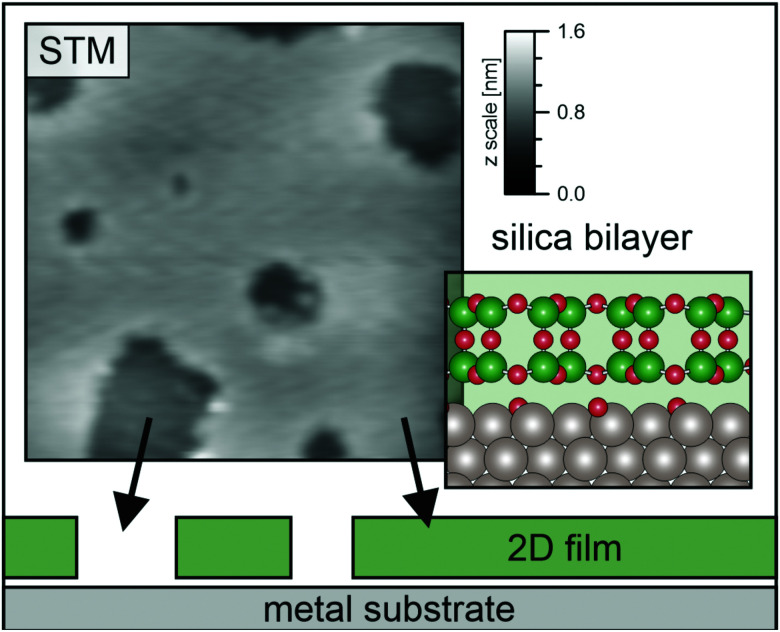
The structure of a silica bilayer film with nanoscale holes. An STM image of the sample used is given in the top left area. The field of view shows 30 nm × 30 nm and a colour coded *z* scale of 1.6 nm (sample voltage *V*_S_ = 1 V, tunnelling current *I*_T_ = 100 pA). A side view of silica bilayer film structure is given by a schema together with a model of the film structure at the bottom area. Si atoms shown as the green, larger, balls, O atoms shown a the smaller red balls. The silica film is supported on a ruthenium substrate. The sample exposes on average 15% holes, which leads to a film coverage of 1.7 ML.

The TOF curves are converted to energy exchange with the surface, Δ*E*. This conversion is shown in Fig. 3, where the TOF spectra seen in the right panel are converted in the left panel. Arrow annotations show phonon peaks. The data points obtained for the dispersion of the ZA-mode for all different azimuthal angles are presented in Fig. 4, along with examples of scan curves shown in the upper panel of the figure. A HAS experiment only sees what lies along its 1D scan curve, and as is seen in Fig. 4, it is possible for the scan curve to intersect with the dispersion relation of the ZA-mode twice. When that happens one expects to find two ZA-mode excitations in the TOF spectrum, which is what is occurring in the red curve shown in Fig. 3. Regarding Fig. 3, note that the weaker peaks are only included if we can track them through several experiments (scan curves) and they can be identified as ZA-modes because they follow the dispersion relation. The ZA-peaks are fitted with the dispersion relation in eqn (2) to obtain the values for the bending rigidity and substrate binding energy that are presented in Table 1. Also listed in Table 1 are the bending rigidities and substrate couplings obtained for the sample investigated in ref. 8. We obtain an average bending rigidity of *κ* = 6.6 ± 0.3 eV and average binding energy of *ω*_0_ = 4 ± 1.5 meV. It is comparatively evident that, while the coupling strength remains similar between the two samples (within error bars), the bending rigidity is smaller with lower sample mass density. The value obtained for *ω*_0_ supports the observations made in ref. 8, and shows that the silica bilayer indeed is weakly bound to the substrate. In turn, this shows that we are within the region where the model in eqn (2) is expected to hold. The binding energy is not expected to change at long wavelengths (short Δ*K*) because the ZA mode is vertically polarised at long wavelengths, with no stretching of the parallel bonds. Therefore, uniformly distributed holes should not impact *ω*_0_. Similar results were obtained for adsorbed rare gas layers on closed-packed metal surfaces, which showed that the ZA-mode frequency for rare gases on metals was essentially independent of the coverage.^29,30^

**Fig. 3:**
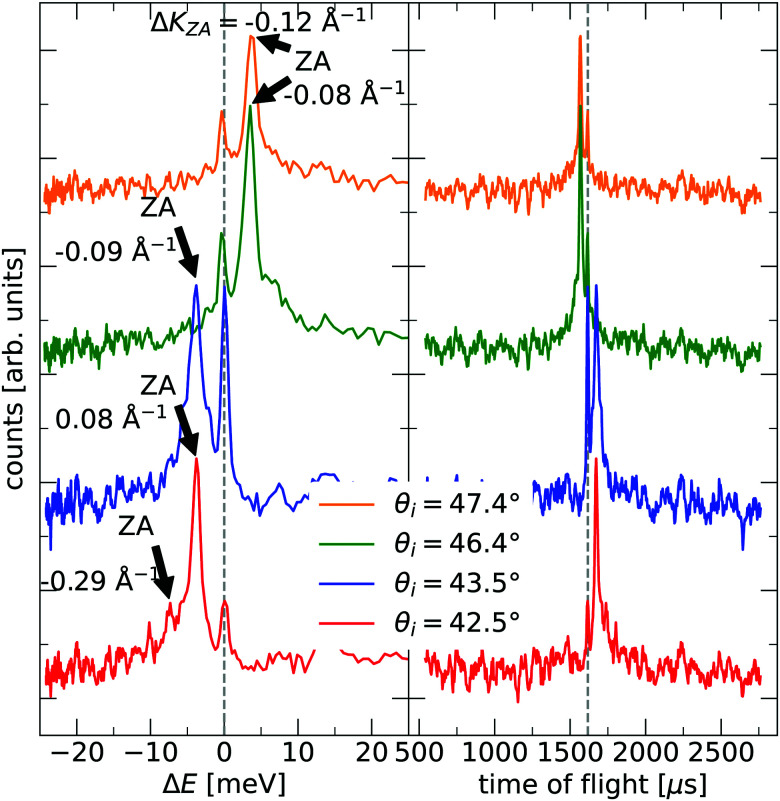
Examples of time of flight measurements (right) and their conversion to energy exchange between the helium atoms and the bilayer (left). Arrow annotations indicate identified ZA-mode peaks in the left panel. Annotations next to the ZA-peaks gives their corresponding Δ*K*-value. Scattering angles are given in descending order, with the first angle corresponding to the upper spectrum. The intensity of the spectra have been arbitrarily shifted to increase visibility.

**Fig. 4:**
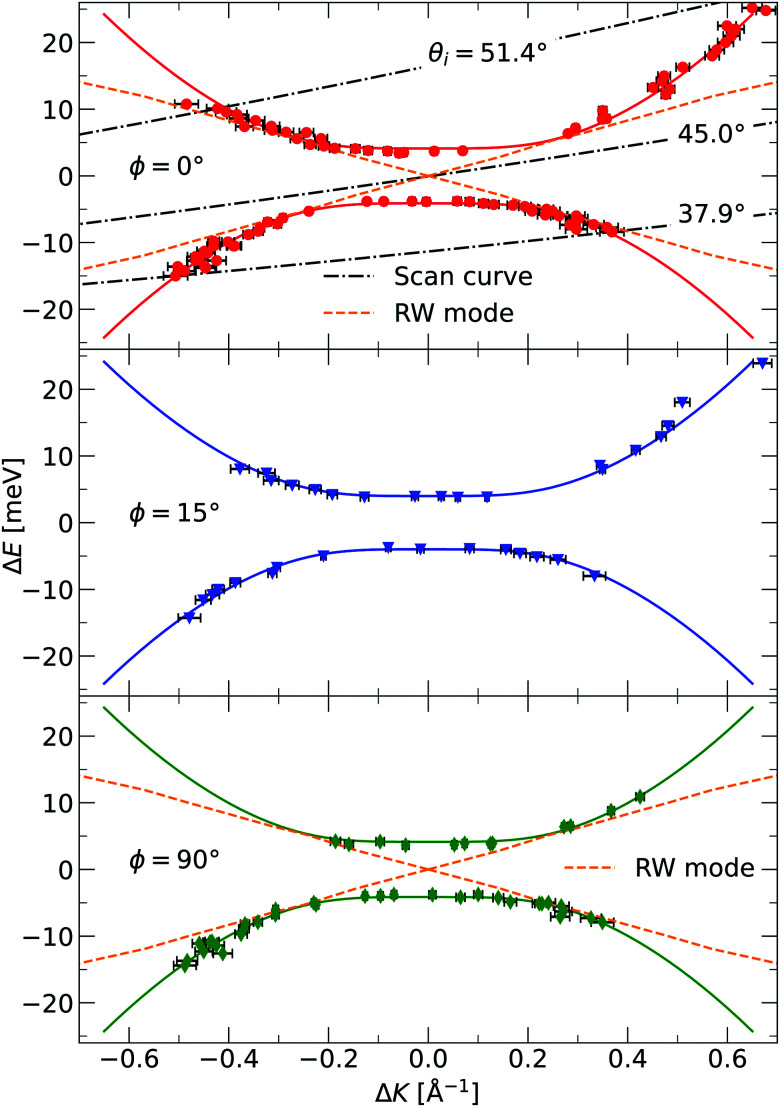
The dispersion relation of the ZA-mode separately plotted for the different azimuthal angles investigated in the experiment, as indicated in each panel. The solid curves are fits of eqn (2) to the identified ZA-modes. Also shown is the dispersion relation for the Rayleigh wave (RW) mode of the Ru(0001) substrate.^28^ The dash-dotted curves in the upper panel are three examples of experimental scan curves, *i.e.* a plot of the energy exchange of the experiment, for a given scattering angle, as a function of parallel momentum transfer. The incident angle is given as an insert in the curve. Error bars are given by read out limitation of the ZA-peak position along the ordinate, and are too small to be seen. The errors in energy are then converted to errors in parallel momentum. The ZA-peak broadens in momentum for larger (in absolute value) parallel momentum transfer, explaining the larger error bars away from Δ*K* = 0.

**Table 1:** Values for bending rigidity *κ* and binding energy *ω*_0_ extracted by fitting eqn (2) to each panel of Fig. 4

Sample	*ρ*_2D_ [mg m^−2^]	*ϕ* [°]	*κ* [eV]	*ω*_0_ [meV]
1 (this study)	1.4	0	6.6 ± 0.2	4 ± 1.5
1	1.4	15	6.6 ± 0.3	4 ± 1.5
1	1.4	90	6.6 ± 0.3	4 ± 1.5
Average	—	—	6.6 ± 0.3	4 ± 1.5

2 (ref. 8)	1.6	0	8.8 ± 0.5	4.2 ± 1.1
2	1.6	17	8.4 ± 1.7	4.3 ± 1.8
2	1.6	90	9 ± 1	4.6 ± 1.6
Average	—	—	8.8 ± 0.5	4.3 ± 1.1

The average value of *κ* obtained for the present sample is 25% lower than the value obtained in ref. 8, with a 14% reduction in sample mass density. With only two different samples, we cannot further claim anything about the empirical relation between the bending rigidity and the sample mass density. Our results do, however, follow recent reviews on the subject. Kim *et al.* recently published a review of the mechanical properties of 2D materials.^31^ They found that point defects in graphene decrease its elastic modulus. The bending rigidity is directly proportional to Young's modulus in the classical picture,^32^ which implies a decrease in bending rigidity. Akinwande *et al.* published another review on the mechanical properties of 2D materials in 2018,^33^ where they conclude that various defects significantly affect the properties of 2D materials. They report that topological defects are likely to decrease the mechanical strength of the membrane.

G. Anemone *et al.* report a decrease in the bending rigidity of graphene on sapphire of about 46% compared to graphene on copper, the latter in the range of values reported for free-standing graphene.^34^ They interpret this result as due to additional defects in the graphene–sapphire system compared to the other two. The defects they observe are nanopits visible along the grain boundaries in atomic force microscopy (AFM) images. López-Polín *et al.* report an increase in Young's modulus of graphene of almost a factor of 2 for a defect density increase up to ≃0.2%.^35^ In the classical picture, the bending rigidity is proportional to Young's modulus,^32^ thus this increase would correspond to an increase in bending rigidity. They introduce mono- and di-vacancy of carbon atoms in their graphene sheets. The authors interpret this result as the vacancies suppressing the out-of-plane fluctuations of the graphene sheet. These defects are different from the nanometer-sized holes present in our bilayer. Topological defects in 2D materials usually refer to vacancies, grain boundaries, and dislocations.^36^ Compared to an ideal, hole-less bilayer, however, a hole can indeed be viewed as a topological defect, as in that the membrane is topologically different, with the number of holes being a topological invariant. A nanometer-sized hole is a topological defect on a length scale different from a vacancy or a grain boundary. The latter two are typically of atomic length scale. The reduction in bending rigidity shows that topological defects of length scales comparable to the material itself are also relevant when discussing its mechanical properties. Another interesting topic is the holes' existence concerning the model in eqn (2), which is valid for a uniform membrane. STM imaging has established that the holes are uniformly distributed throughout the membrane, and they are, on average, considerably smaller (in diameter) than the coherence length of the helium beam. One, therefore, expects that the model works, which is confirmed by the excellent agreement between the data and the model in Fig. 4. In the investigated momentum-energy space area, the Rayleigh wave (RW) of the Ru(0001) substrate might be expected to be visible through the silica bilayer. The predicted position of this mode can be seen in the upper and lower panels of Fig. 4, and although it merges with the measured points of the ZA-mode, there is no visible mode in the areas where the curves do not intersect. Especially in the area close to the origin for |Δ*E*| < 4 meV, we should be able to observe the RW-mode if it was visible through the bilayer. The lack of observation leads us to conclude that even if the RW-mode should be visible through the bilayer, it is much weaker than the ZA-mode and has a negligible impact on the measurements.

We have applied inelastic HAS to measure ZA mode excitation to a silica bilayer film with nanoscale holes. Measuring the ZA mode over an extensive range of experimental incident angles allowed us to fit a dispersion relation to the ZA mode excitation and extract the bending rigidity of the bilayer film and the binding energy between the bilayer silica and the substrate. Compared with a silica sample with higher mass density, the bending rigidity decreases with lower sample mass density, whereas the binding energy remains unchanged. Future work should include more measurements of membranes with other densities to establish a relation between the bending rigidity and mass density. Furthermore, it would be interesting to test the model's validity by deliberately decreasing the coherence length of the helium beam through a decrease in angular resolution.

## Conflicts of interest

There are no conflicts to declare.

## Supplementary Material
